# Reducing health risk in family members of patients with type 2 diabetes: views of first degree relatives

**DOI:** 10.1186/1471-2458-9-455

**Published:** 2009-12-10

**Authors:** David L Whitford, Hannah McGee, Bernadette O'Sullivan

**Affiliations:** 1 Department of Family Medicine, Royal College of Surgeons in Ireland - Medical University of Bahrain, PO Box 15503, Adliya, Kingdom of Bahrain; 2 Department of Psychology, Population Health Sciences, Royal College of Surgeons in Ireland, Dublin 2, Ireland

## Abstract

**Background:**

Patients with type 2 diabetes can have an important role in discussing health risk within families. This study aimed to establish the acceptability to first degree relatives towards their relative with type 2 diabetes intervening as health promoters in their own families, using the Health Belief Model as a theoretical framework for evaluation.

**Methods:**

Cross-sectional questionnaire design. Survey questionnaire for first degree relative (sibling or child) mailed to a random sample of patients with type 2 diabetes registered with an urban hospital diabetes clinic (n = 607 eligible patients). Patients were asked to pass on questionnaires to one to two first degree relatives.

**Results:**

Questionnaires were returned from 257 families (42% response rate) with two responses provided by 107 families (a total of 364 questionnaires). The majority (94%) of first degree relatives of patients with type 2 diabetes would like to be informed about reducing their risk. Half (48%) of respondents reported being spoken to by a relative with type 2 diabetes about their risk of diabetes. Those spoken to were more likely to see themselves at risk of diabetes, to worry about developing diabetes and to view diabetes as a serious condition.

**Conclusions:**

A role for patients with type 2 diabetes in discussing health risk in their family appears to be acceptable to many relatives. Discussion of risk and interventions to reduce health risk with their relatives should be encouraged in patients with type 2 diabetes.

## Background

First degree relatives and spouses of individuals with type 2 diabetes are a group at increased risk of developing type 2 diabetes[1,2] Maternal family history (FH) confers a relative risk of 2.0 to 3.4, combined maternal and paternal FH of 2.6 to 6.1, paternal FH of 1.4 to 3.5, and sibling FH a relative risk of 1.8[1]. This is equivalent to a 20-40% absolute risk in children of one parent with type 2 diabetes[3]. A positive family history has been shown to have a higher positive predictive value for diabetes than obesity[4,5] (There is also an established correlation of other cardiovascular risk factors in family members including obesity[6], hypertension[7], lipids[8] and smoking[8]. This increased family risk is thought to have both a genetic and environmental basis[9,10], giving scope for decreasing the cardiovascular risk through lifestyle modification in individuals with a family history of diabetes[11]. Nonetheless, systematic screening of family members is unlikely for logistic and financial reasons. A more modest approach would be to encourage patients with diabetes to discuss risk with family members.

According to the Health Belief Model[12], engaging family members in such a preventive activity is determined by key factors: perceived susceptibility, perceived severity, perceived benefits, perceived barriers and cues to action. Furthermore, it is suggested that the extent to which individuals see their health to be under their own influence may also be a factor in health decisions[13]. Hence, two other variables frequently added to studies including the Health Belief Model are the value the individual places on health and the individual's health locus of control beliefs, i.e. whether individuals consider their health to be under the control of internal factors, powerful others, or chance.

Involving patients in counselling their families has implications for many genetic diseases[14]. However, previous studies have shown that children of individuals with type 2 diabetes underestimate their risk of diabetes, have a poor sense of the seriousness of type 2 diabetes and know little about reduction of their risk[15]. Patients with type 2 diabetes recognise the need to give preventive health advice to family members but do not necessarily carry this out[16,17]. This study aims to establish the acceptability to first degree relatives of their relative with type 2 diabetes intervening as health promoters in their own families. We used the Health Belief Model as a predictor of the possibility of first degree relatives being engaged in the prevention of diabetes by their relative with diabetes.

## Methods

### Sample

**Figure 1:**
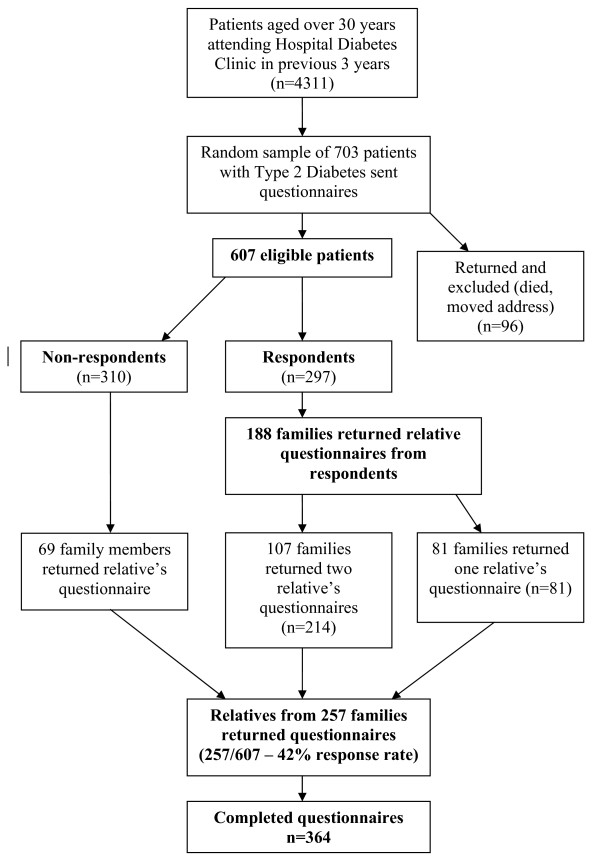
**Sampling and Responses**.

There is no readily available method to identify all members of the population of interest (first degree relatives of patients with type 2 diabetes). In this study we used patients with type 2 diabetes involved in an associated study[18] as the basis for sampling. This patient population was a random sample of patients with type 2 diabetes (n = 607 eligible patients) registered with a hospital diabetes clinic (Figure 1). Following hospital Research Ethics Committee approval, these patients with diabetes were asked if they would pass on an invitation to participate in a postal survey to one or two of their first degree relatives (i.e. brother, sister or child) aged over 18 years who were capable of completing the questionnaire. They were asked to exclude first degree relatives who had diabetes themselves, who lived outside of Ireland or who did not know that the patient had diabetes. While this method may introduce a selection bias, it provides a reasonable method of sampling a hard-to-reach group. Since consent to participate was a two-phase process (willingness of patient with diabetes to pass on the questionnaire and willingness of relatives to complete and return the questionnaire), this method would not provide information on the numbers of questionnaires passed on to relatives as compared to the number returned. The initial mailing in mid 2006 was followed by a reminder after a further three weeks. The Research Ethics Committee accepted return of a completed questionnaire as provision of informed consent.

### Questionnaire development

We developed a questionnaire to assess the perceptions of first degree relatives of patients with type 2 diabetes about prevention of type 2 diabetes, based on the parameters of the Health Belief Model. Initial questions were adapted from a previous study[15], e.g. on knowledge of complications. The primary endpoint was the response to a question asking 'If my relative with diabetes received information about how I could possibly reduce my risk of getting diabetes, I would like him or her to talk to me about how I could do this'. Relatives responded on a six point Likert scale from 'Strongly disagree' to 'Strongly agree'. A secondary endpoint was the categorical response to the question 'Has your relative with diabetes talked to you about the possibility of getting diabetes?' In addition, three focus groups attended by 17 relatives of patients with type 2 diabetes (11 daughters, 3 sons, and 3 sisters) were conducted to identify themes concerning benefits and barriers towards prevention of type 2 diabetes. Questions were developed from these themes and incorporated into the questionnaire. Further questions explored perception of family risk of diabetes, anxiety about diabetes in the family, knowledge of diabetes risk factors, perceptions of the seriousness of diabetes and of prevention of diabetes. Further established measures were also incorporated. These included the Health Value Scale in order to assess the value of health as a possible motivator of preventive health behaviour[19] and the Diabetes Onset Locus of Control scale[20] to assess perceived control over the condition. Background information was collected on the respondents' age, gender, marital status, educational status, family history of diabetes and year of diagnosis of their relative with diabetes. The questionnaire was piloted and refined [see Additional File 1]. A similar questionnaire on the views of family members with diabetes had been previously developed[18]. Both questionnaires from patients with diabetes and their relatives were given unique ID numbers in order to identify family groups for analysis.

### Data analysis

Data were analysed using descriptive statistics. Data analysis was with SPSS 14.0 using parametric and non-parametric tests of association. In order to investigate clustering of knowledge and attitudes within families, paired analysis of relatives' responses and the responses from patients with type 2 diabetes to an associated questionnaire was also performed. P < 0.05 was taken as significant.

## Results

There were 607 eligible patients from the sample of 703 patients with diabetes (Figure 1). Relatives of 257 of these patients with diabetes returned questionnaires (42% response rate). There were replies from two members of 107 (18%) families. Thus there were a total of 364 completed relative questionnaires (Figure 1). The mean age of respondents was 44 years (range 18-83 years) and 34% were male. Respondents were given the questionnaire by their father (44%), mother (28%), or sibling (24%). 15% had more than one first degree relative with type 2 diabetes. The relatives of respondents had diabetes for a mean of seven years and were treated with diet alone (18%), tablets (60%) or insulin (22%).

### Perceived Susceptibility and Seriousness

Respondents were asked to rate how likely it was that they would develop diabetes sometime in their life (susceptibility). Over half (55%) thought it not at all or not very likely that they would develop diabetes (Table 1). 63% thought that it was unlikely a person would develop diabetes if they had no family history of diabetes.

**Table 1:** Perceptions of diabetes and health-related cognitions

Health Related Cognition	Measure	Results
**Personal susceptibility**	**Likelihood will develop diabetes**	**Number (%)**
	Not at all	22 (6)
	Not very	169 (50)
	Quite likely	13 (38)
	Very likely	20 (6)

**Perceived seriousness**	**Disease**	**Scale (95% CI)**
	(Scale 1-5 from 'not at all serious' to 'very serious')	
	Cancer	4.91 (4.88-4.95)
	AIDS	4.87 (4.83-4.92)
	Diabetes	4.01 (3.91-4.11)
	Arthritis	3.78 (3.68-3.88)
	Flu	2.07 (1.96-2.19)

**Anxiety**	**Worries may develop diabetes**	**Number (%)**
	Never	101 (30)
	Rarely	74 (22)
	Sometimes	127 (37)
	Often	36 (11)

**Health Value Scale**	Scores 4-28. Higher scores indicate a higher value on health.	**Score (95% CI)**
		23.3 (22.8-23.8)

**Diabetes Onset Locus of Control**	**Scale**	**Score (95% CI)**
	(Subscale range 0-20; maximum overall score 60)	
	Overall score	37.6 (36.5-38.7)
	Powerful others (e.g. health professionals)	16.5 (16.0-16.9)
	Significant others (e.g. family and friends)	12.0 (11.6-12.4)
	Internality	18.6 (18.2-19.0)
	Chance	9.5 (9.0-9.9)

**Benefits of adopting healthy lifestyle**	**Measure**	**% Moderately or Strongly Agreeing**
	I would reduce my chances of getting diabetes (n = 353)	96
	I would reduce my risk of other diseases, such as heart disease (n = 354)	96
	I would feel fit and healthy (n = 354)	98
	I would keep my weight under control (n = 356)	98

**Barriers to adopting healthy lifestyle**	**Measure**	**% Moderately or Strongly Agreeing**
	I do not have time to exercise (n = 353)	66
	I do not like the taste of low-fat food (n = 355)	71
	I do not have time to prepare healthy food (n = 354)	74
	I would find it difficult to motivate myself to exercise (n = 354)	55

Respondents were then asked to rate the seriousness of five conditions, including diabetes, on a 5-point scale. The rank order of the five conditions from most to least serious was cancer, AIDS, diabetes, arthritis and flu (Table 1).

### Diabetes Onset Locus of Control, Health Value and Anxiety

A locus of control orientation is a belief about whether the outcomes of our actions are contingent on what we do (internal control orientation) or on events outside our personal control (external control orientation). The Diabetes Onset Locus of Control (DOLoC) questionnaire is designed to assess this belief in individuals concerning their development of diabetes[20]. Higher total scores indicate a high degree of control for self and others in determining development of diabetes. The mean score for total diabetes onset locus of control was 37.6 (95%CI 36.5-38.7). Results for overall diabetes onset locus of control and for subscales are shown in Table 1. Scores for respondents indicate a high degree of control for self and powerful others (e.g. health professionals) in determining the development of diabetes.

The Health Value Scale is a measure of the value an individual places on his/her health, with a range of scores from 4-28. Higher scores indicate a higher value on health. Scores for respondents indicate a high value placed on health[19] in this sample (Table 1).

Half of respondents stated that they often or sometimes worried about developing diabetes at some point in their life.

### Knowledge of Risk Factors for Diabetes

Respondents were asked which of a list of factors increased a person's risk of type 2 diabetes; 80% identified obesity and 58% identified taking little or no exercise. However, only 52% identified having a parent with diabetes as increasing the risk of diabetes and only 29% identified having a sibling as conferring an increased risk, while 30% chose 'high salt intake' as giving an increased risk of diabetes.

### Perceived Benefits and Barriers

Table 1 shows the responses to perceived benefits and barriers of adopting a healthy diet and regular physical activity. Respondents were very convinced about the benefits of diet and physical activity but over half identified time and lack of motivation as challenges to them in engaging in physical activity and a higher proportion identified lack of time and inclination to prepare low fat foods.

### Desire to be informed of Health Risk

The dependent variable in this study was the desire to be spoken to about the possibility of reducing their risk of diabetes. Overall, 6% of respondents disagreed that they would like to be spoken to by a relative with diabetes about reducing their risk of diabetes. There was no difference across demographic indices between those who would like a relative to speak to them and those who would not (Table 2).

**Table 2:** Relationship between respondent characteristics & views with whether they would like to be informed by relatives as to reduction of their health risk

	*"If my relative with Type 2 Diabetes received information about how I could possibly reduce my risk of getting diabetes, I would like him or her to talk to me about how I could do this."*			
**Variable**		**Disagree n (%)**	**Agree n (%)**	**Statistic**

Total (n = 321)		19 (6)	302 (94)	

Gender (n = 321)	Male	5 (5)	101 (95)	X^2 ^= 0.41, 1 df, p = 0.52
		
	Female	14 (6.5)	201 (93.5)	

Age (n = 318)	< 40 years	10 (7)	139 (93)	X^2 ^= 0.61, 2 df, p = 0.74
		
	40-49 years	6 (6)	90 (94)	
		
	> 49 years	3 (4)	70 (96)	

Marital status (n = 319)	Single/widowed	4 (4)	98 (96)	X^2 ^= 0.83, 1 df, p = 0.36
		
	Married/cohabiting	14 (6)	203 (94)	

Age finished full-time education (n = 306)	< 17 years	5 (6)	80 (94)	X^2 ^= 0.06, 2 df, p = 0.97
		
	17-18 years	8 (6)	121 (94)	
		
	> 18 years	5 (5)	87 (95)	

View on likelihood of developing diabetes in life (n = 315)	Not at all/Not very likely	11 (8)	125 (92)	X^2 ^= 1.79, 1 df, p = 0.18
		
	Quite/Very likely	8 (4)	171 (96)	

View on likelihood of developing diabetes if no family history (n = 316)	Not at all/Not very	13 (7)	182 (93)	X^2 ^= 0.38, 1 df, p = 0.53
		
	Quite/Very likely	6 (5)	115 (95)	

Worries that may get diabetes (n = 316)	No/Rarely	10 (6)	155 (94)	X^2 ^= 0.001, 1 df, p = 0.97
		
	Sometimes/Often	9 (6)	142 (94)	

View on seriousness of diabetes to cancer (n = 310)	Diabetes as serious	0	109 (100)	X^2 ^= 10.36, 1 df, p = 0.001
		
	Diabetes less serious	18 (9)	183 (91)	

View on seriousness of diabetes to arthritis (n = 309)	Diabetes more serious	10 (7)	126 (93)	X^2 ^= 3.22, 2 df, p = 0.2
		
	Diabetes as serious	5 (8)	56 (92)	
		
	Diabetes less serious	3 (3)	109 (97)	

Has been spoken to by family member about possibility of getting diabetes (n = 309)	Yes	5 (3)	139 (97)	X^2 ^= 3.35, 1 df, p = 0.07
		
	No	14 (8)	151 (92)	

Respondents who disagreed that they would like a relative with diabetes to speak to them about reducing their risk of diabetes were less likely to regard diabetes as being as serious as cancer. There was no association with Diabetes onset Locus of Control or with the value placed on health.

Half (48%) of respondents had been spoken to by a relative with type 2 diabetes about their risk of diabetes (Table 3). They were more likely to see themselves at risk of diabetes (X^2 ^= 9.57, 1 df, p = 0.002), more likely to worry about developing diabetes (X^2 ^= 5.9, 1 df, p = 0.02) and more likely to see diabetes as more serious than arthritis (X^2 ^= 11.4, 2 df, p = 0.003).

**Table 3:** Characteristics of respondents and whether they had been spoken to by a family member about the possibility of developing diabetes

Characteristic	Values	Number spoken to about risk of diabetes (%)	Statistic
Age (n = 322)	< 40 years (n = 150)	90 (60)	X^2 ^= 17.77, 2 df, p < 0.001
		
	40-49 years (n = 97)	34 (35)	
		
	> 49 years (n = 75)	29 (39)	

Gender (n = 326)	Male (n = 105)	43 (41)	X^2 ^= 2.7, 1 df, p = 0.10
		
	Female (n = 221)	113 (51)	

Marital status (n = 324)	Single/widowed (n = 98)	56 (57)	X^2 ^= 4.87, 1 df, p = 0.027
		
	Married/cohabiting (n = 226)	99 (44)	

Occupation (n = 288)	Management/Professions (n = 82)	36 (44)	X^2 ^= 13.47, 3 df, p = 0.004
		
	Skilled workers (n = 78)	34 (44)	
		
	Unskilled workers (n = 46)	33 (72)	
		
	No employment (n = 82)	33 (40)	

Number of first degree relatives with diabetes (n = 307)	One (n = 261)	130 (50)	X^2 ^= 4.85, 1 df, p = 0.028
		
	More than one (n = 46)	15 (33)	

Relative who gave questionnaire (n = 310)	Father (n = 137)	68 (51)	X^2 ^= 9.09, 3 df, p = 0.028
		
	Mother (n = 88)	47 (53)	
		
	Sibling (n = 74)	26 (35)	
		
	Other (n = 11)	8 (73)	

Likely to develop diabetes in life (n = 321)	Not at all/Not very likely (n = 140)	53 (38)	X^2 ^= 9.57, 1 df, p = 0.002
		
	Quite/Very likely (n = 181)	100 (55)	

Likely to develop diabetes if no family history (n = 320)	Not at all/Not very (n = 198)	81 (41)	X^2 ^= 9.92, 1 df, p = 0.002
		
	Quite/Very likely (n = 122)	72 (59)	

Worry that may get diabetes (n = 320)	No/Rarely (n = 167)	68 (41)	X^2 ^= 5.9, 1 df, p = 0.015
		
	Sometimes/Often (n = 153)	84 (55)	

Ratio seriousness diabetes to arthritis (n = 314)	Diabetes more serious (n = 118)	70 (59)	X^2 ^= 11.4, 2 df, p = 0.003
		
	Diabetes as serious (n = 133)	53 (44)	
		
	Diabetes less serious (n = 63)	22 (35)	

Ratio seriousness diabetes to cancer (n = 315)	Diabetes as serious (n = 110)	55 (50)	X^2 ^= 0.29, 1 df, p = 0.59
		
	Diabetes less serious (n = 205)	96 (47)	

Knowledge that parent with T2D is risk (n = 326)	Yes (n = 174)	99 (57)	X^2 ^= 13.08, 1 df, p < 0.001
		
	No (n = 152)	56 (37)	

Knowledge that sibling with T2D is risk (n = 326)	Yes (n = 96)	52 (54)	X^2 ^= 2.39, 1 df, p = 0.12
	No (n = 230)	104(45)	

### Paired Analysis of Patient and Relative Responses

Paired data from the relative's questionnaire and one completed by patients with type 2 diabetes as part of an associated study[18] was available for 295 relatives. There was no association between the gender of patients with type 2 diabetes and the gender of the relatives who received questionnaires (X^2 ^= 0.76, 1 df, p = 0.38). Patients with greater knowledge about risk factors for type 2 diabetes were more likely to be related to respondents with greater knowledge of these risk factors (e.g. having a parent with type 2 diabetes (X^2 ^= 20.4, 1 df, p < 0.001), taking little exercise (X^2 ^= 7.39, 1 df, p = 0.006), being over 40 years of age (X^2 ^= 12.38, 1 df, p < 0.001), having a sibling with type 2 diabetes (X^2 ^= 13.44, 1 df, p < 0.001), high salt intake not a risk (X^2 ^= 9.3, 1 df, p = 0.002)). Perceptions of susceptibility to type 2 diabetes were also shared between patients and their relatives. Patients who thought their children were more likely to get type 2 diabetes were more likely to have relatives who believed they were at greater risk of developing type 2 diabetes (X^2 ^= 11.0, 1 df, p < 0.001). However, patients who thought their siblings were more likely to get type 2 diabetes were not more likely to have relatives who believed they were at higher risk of type 2 diabetes (X^2 ^= 0.02, 1 df, p = 0.89). Patients who worried more about their children developing type 2 diabetes were more likely to have relatives who worried about developing type 2 diabetes themselves (X^2 ^= 6.94, 1 df, p = 0.008).

Families were also likely to perceive the seriousness of diabetes similarly. Patients and their relatives were more likely to have similar perceptions of the seriousness of diabetes as compared with cancer (X^2 ^= 10.92, 1 df, p = 0.001) and arthritis (X^2 ^= 13.24, 4 df, p = 0.04). They were also more likely to place similar value on their health (t = 3.45, p < 0.001). Relatives who strongly agreed with being spoken to about what they could do to prevent developing type 2 diabetes were more likely to be related to a patient with type 2 diabetes who strongly agreed with being trained to inform relatives of preventative strategies for type 2 diabetes (X^2 ^= 16.7, 4 df, p = 0.002).

## Discussion

This study shows that relatives of patients with type 2 diabetes who have been spoken to about their risk of diabetes have an increased perception of their susceptibility to the disease, an increased sense of its seriousness, an increased knowledge of risk factors and greater anxiety about developing diabetes. The majority of relatives of patients with type 2 diabetes would like to be informed about reducing their health risk. The respondents who disagreed with this had less appreciation of the 'threat' (susceptibility and seriousness) of type 2 diabetes. Emphasising threat of diabetes and associated cardiovascular risk would provide a theoretical basis for encouraging patients to inform family members about reducing their health risk.

Patients who were more willing to inform relatives of their health risk were related to respondents who were more encouraging towards this approach. In addition, it is not surprising (but very encouraging) that patients and their relatives had similar attitudes, knowledge, and anxiety about the development of type 2 diabetes within their family. This suggests that proactively encouraging patients with type 2 diabetes to communicate with their relatives about health risk and strategies to reduce that risk would positively influence knowledge and attitudes and hopefully lead to changes in health behaviours.

This study also reveals an encouraging improvement in knowledge and attitudes towards family risk of type 2 diabetes among relatives of patients with type 2 diabetes compared with previous studies. For instance, compared with a study six years previously in the UK[15], this study suggests that there has been an increase in awareness amongst relatives of their susceptibility to type 2 diabetes (46% thinking it likely or very likely that they would develop diabetes in this study compared with 34% in the UK study); an increase in worry that they would develop type 2 diabetes (49% in this study, 28% in the UK study); and an increase in the number of relatives who had been spoken to about their risk of developing type 2 diabetes (48% in this study, 30% in the UK study). This was associated with an increased knowledge of the risk factors for type 2 diabetes (e.g. comparing this study with the UK study: knowledge of parent as risk factor - 52% vs. 49%; knowledge of sibling as risk factor - 29% vs. 8%; knowledge of obesity as a risk factor - 80% vs. 38%; and knowledge of lack of physical activity 58% vs. 21%). In spite of these higher levels of awareness, it is clear that awareness of family history as a risk factor for diabetes is far from optimal, especially as this is a selected sample of relatives who are more likely to be more aware than others.

In terms of study limitations, it was not possible to identify a population sampling frame of relatives and therefore not possible to obtain a randomised sample. The chosen sampling method introduced a selection bias, with the likelihood that the patients with type 2 diabetes would distribute the questionnaire to relatives they were in contact with and thus more likely to have spoken to about their diabetes. They may also have provided questionnaires to those relatives most interested in their own diabetic condition. The low response rate may be related to the sampling method and targeting a population that may be less inclined to respond, but nevertheless reduces generalisability of the results. In addition, in order to increase study participation, two questionnaires were given to each patient with an invitation to invite 'one to two' family members to respond. This introduced a clustering effect which may reduce variability in results. However, the study reflects the views of family members that are in contact with their relative with type 2 diabetes. A more proactive approach towards encouraging patients with diabetes to inform relatives of their health risk would most likely have its greatest impact in this population of relatives. Another point of note was the preponderance of women among respondents, both those participating in focus groups and in responders to the survey. There were no gender differences among those included in the study in outcomes. However, this selection bias means that these similarities across men and women cannot be assumed to be generalisable. A strength of this study is the use of a theoretical model (the Health Belief Model) in designing the questionnaire; ensuring parameters likely to influence preventive health behaviours in the family were addressed.

In the search for more cost-effective routes to the reduction of cardiovascular risk in a population, those with a familial risk of type 2 diabetes have been proposed as a possible high risk group in which to intervene[11]. As reported elsewhere[18], three quarters of patients with type 2 diabetes were willing to take on this health-promoting role within families. This study indicates that relatives of patients with type 2 diabetes are also supportive of being informed by their relatives of their risk and of strategies to help reduce this risk. Future studies should stratify sub-groups to determine those groups who would be more or less engaged in terms of giving or accepting this preventive health message. Stratification - by relationship of relative to patient with diabetes (child vs. sibling); by gender of both patient and relative; and by clinical status and duration of diabetes (more/less serious clinical status and shorter vs. longer of diabetes) - would all refine efforts to promote a preventive approach among families.

## Conclusions

One strategy to prevent diabetes and associated cardiovascular risk is to intervene in families with a high risk of type 2 diabetes. This study suggests that this option is becoming more realistic with a growing sense among first degree relatives of the seriousness of diabetes, of their susceptibility to diabetes and a desire to act on these concerns. Discussion of risk and interventions to reduce health risk with their relatives should be encouraged in patients with type 2 diabetes.

## Competing interests

The authors declare that they have no competing interests.

## Authors' contributions

DW conceived of the study, and participated in its design, coordination and statistical analysis and drafted the manuscript. HM participated in the design of the study and drafting of the manuscript. BOS participated in the design of the study, data collection and statistical analysis. All authors read and approved the final manuscript.

## Pre-publication history

The pre-publication history for this paper can be accessed here:

http://www.biomedcentral.com/1471-2458/9/455/prepub

## Supplementary Material

Additional file 1 **Questionnaire**. Copy of questionnaire used in the study. Click here for file
