# Supplementary material for: Reducing health risk in family members of patients with type 2 diabetes: views of first degree relatives
Source: BMC Public Health. 2009 Dec 10;9:455. doi: 10.1186/1471-2458-9-455 (PMC2796668; doi:10.1186/1471-2458-9-455)
Supplement: Additional file 1 — Questionnaire. Copy of questionnaire used in the study. [file 1471-2458-9-455-S1.DOC]

Code:

# questionnaire

### Section 1

*There is a question on diabetes below. It is followed by a number of choices. You should select from these choices* ***one or more*** *that you think correctly answers the question. Do not worry if you cannot answer the question, just tick the box next to “I don’t know”. Please do not try finding out the answer before you complete this section or try guessing the answer*.

For example,

Most people normally travel to and from work by…

Bus/Train **√**

Horse

Car/Motorcyle **√**

Bicycle **√**

Airplane

# I don’t know

1. Which of the following factors make a person more likely to develop type 2 diabetes?

Having a parent with type 2 diabetes

Being overweight

High salt intake

Taking little or no exercise

Being over 40 years of age

Having a brother or sister with type 2 diabetes

I don’t know

Section 2

*Please circle the number that represents your answer to the following questions. For example, if you thought that something was quite likely, you would circle the number 3.*

###### Not at all Not very Quite Very

likely likely likely likely

1. How likely do you think it is that you

will get diabetes sometime in your life? 1 2 3 4

###### 2. How likely do you think it is that

###### someone will get diabetes if he or she

###### does not have a family history of diabetes? 1 2 3 4

###### No Rarely Sometimes Often

3. Do you worry that you might get

diabetes sometime in your life? 1 2 3 4

# Section 3

This section is designed to determine the way in which different people view he possibility of developing diabetes. Each item is a belief statement with which you may agree or disagree. Beside each statement is a scale, which ranges from strongly disagree (1) to strongly agree (6). For each item we would like you to circle the number that represents the extent to which you disagree or agree with the statement. The more strongly you agree with a statement, then the higher will be the number you circle. The more strongly you disagree with the statement, then the lower will be the number you circle. Please make sure that you answer every item and that you circle only one number per item. This a measure of your personal beliefs; there are, therefore, no right or wrong answers.

Please answer these items carefully, but do not spend too much time on any one item. As much as you can, try to respond to each item independently. When making your choice, do not be influenced by your previous choices. It is important that you respond according to your actual beliefs and not according to how you feel you should believe or how you think we want you to believe.

# Strongly Moderately Slightly Slightly Moderately Strongly

Disagree Disagree Disagree Agree Agree Agree

1. Whether or not I get diabetes

depends largely on my own efforts. 1 2 3 4 5 6

2. My work and/or domestic

situation has a large influence on

whether I develop diabetes. 1 2 3 4 5 6

3. If I get diabetes it won’t be

because of other people or

circumstances. 1 2 3 4 5 6

# Strongly Moderately Slightly Slightly Moderately Strongly

Disagree Disagree Disagree Agree Agree Agree

4. Developing diabetes is largely a

matter of bad luck. 1 2 3 4 5 6

5. Even those closest to me would

not be able to influence whether or

not I develop diabetes. 1 2 3 4 5 6

6. If I am going to get diabetes

there is nothing the medical

profession can do to prevent it. 1 2 3 4 5 6

7. I do not believe in the role of

fate as to whether or not I develop

diabetes. 1 2 3 4 5 6

8. The best way to try to avoid

developing diabetes is to make it

my own responsibility. 1 2 3 4 5 6

9. I can probably avoid developing

diabetes by getting the right

medical advice. 1 2 3 4 5 6

10. I’m not likely to be able to

influence the risk of my developing

diabetes. 1 2 3 4 5 6

11. Nobody knows enough to

ensure that I stay free of diabetes. 1 2 3 4 5 6

12. No matter how much effort I

put in I cannot influence whether

or not I get diabetes. 1 2 3 4 5 6

13. Fate will determine whether

or not I develop diabetes. 1 2 3 4 5 6

14. I do not think that other people

in my life will affect whether or not

I stay free of diabetes. 1 2 3 4 5 6

# Strongly Moderately Slightly Slightly Moderately Strongly

Disagree Disagree Disagree Agree Agree Agree

15. Doctors or nurses cannot

influence whether or not I develop

diabetes. 1 2 3 4 5 6

16. Avoiding diabetes has nothing

to do with luck. 1 2 3 4 5 6

Section 4

*Please circle a number on each of the scales to indicate how serious you think the following problems are.*

Not Mildly Quite Very

serious serious serious Serious serious

1. Cancer 1 2 3 4 5
2. Flu 1 2 3 4 5
3. Diabetes 1 2 3 4 5
4. AIDS 1 2 3 4 5

5. Arthritis 1 2 3 4 5

# Section 5

*Indicate the extent to which you agree with the following four statements, using the scale below. Write the appropriate number in the blank space to the right of each statement*.

Strongly Moderately Moderately Strongly

agree agree disagree disagree

1 2 3 4 5 6 7

1) There is nothing more important than good health. ______

2) Good health is only of minor importance in a happy life. ______

3) If you don’t have your health, you don’t have anything. ______

4) There are many things I care about more than my health. ______

# Section 6

Eating a healthy low-fat diet and regular physical activity of moderate intensity (e.g. brisk walking) have been identified as two possible strategies to reduce a person’s risk of getting diabetes. Listed below are some possible benefits and barriers related to these lifestyle choices. *Please circle the number that represents the extent to which you disagree or agree with each statement.*

# Strongly Moderately Slightly Slightly Moderately Strongly

Disagree Disagree Disagree Agree Agree Agree

Benefits

1. I would reduce my chances of

getting diabetes. 1 2 3 4 5 6

2. I would reduce my risk of

developing other diseases,

such as heart disease. 1 2 3 4 5 6

3. I would feel fit and healthy. 1 2 3 4 5 6

4. I would keep my weight under

control. 1 2 3 4 5 6

# Strongly Moderately Slightly Slightly Moderately Strongly

Disagree Disagree Disagree Agree Agree Agree

Barriers

1. I do not have time to exercise. 1 2 3 4 5 6

2. I do not like the taste of

low-fat food. 1 2 3 4 5 6

3. I do not have the time to

prepare healthy low-fat food. 1 2 3 4 5 6

4. I would find it difficult to

motivate myself to exercise. 1 2 3 4 5 6

# Section 7

The questions in this section relate to your family history of diabetes. Please indicate if your mother or father or any of your brothers or sisters has/had diabetes by ticking the “Yes”, “No” or “I don’t know” box.

|  | Yes | No | I don’t know |
| --- | --- | --- | --- |
| 1. My mother |  |  |  |
| 2. My father |  |  |  |
| 3. My brothers and sisters |  |  |  |

Section 8

1. We asked one of your relatives, who has Type 2 diabetes, to give you this questionnaire. Please write down in the space provided what relation this person is to you (e.g. my mother). _____________________

2. When was this person diagnosed with diabetes?

Month ________Year ______ I don’t know

3. How is his or her diabetes treated? Please tick the box next to your answer.

Diet Diet + tablets Diet + insulin Diet, tablets, + insulin

I don’t know

4. Has this person talked to you about the possibility of you getting diabetes?

Yes No

5. *Please circle the number that corresponds to your level of agreement with the following statement.*

If this person received information about how I could possibly reduce my risk of getting diabetes, I would like him or her to talk to me about how I could do this.

Strongly Moderately Slightly Slightly Moderately Strongly

disagree disagree disagree agree agree agree

1 2 3 4 5 6

# Section 9

1. Are you male or female?

2. What age are you? _____

3. Are you married/living with your partner widowed separated/divorced single?

4. At what age did you finish fulltime education? _____

5. What is your occupation? *If you are retired, please write this down as well as your previous occupation. If you are a homemaker/housewife, please write this down as well as the occupation of the main breadwinner in your household.*

_______________________________________________________________________________

**Thank you very much for taking the time to complete this questionnaire.**
